# *cis*-[1,4-Bis(diphenyl­phosphan­yl)butane-κ^2^ *P*,*P*′]dichlorido(cyclo­hexane-1,2-diamine-κ^2^ *N*,*N*′)ruthenium(II) dichloro­methane monosolvate

**DOI:** 10.1107/S1600536812014080

**Published:** 2012-04-13

**Authors:** Ismail Warad

**Affiliations:** a Department of Chemistry, AN-Najah National University, Nablus, Jordan

## Abstract

In the title compound, [RuCl_2_(C_6_H_14_N_2_)(C_28_H_28_P_2_)]·CH_2_Cl_2_, the Ru^II^ ion is coordinated in a slightly distorted octa­hedral environment, formed by two *cis*-oriented chloride ligands, two *cis* P atoms of a 1,4-bis­(diphenyl­phosphan­yl)butane ligand and two *cis*-chelating N atoms of a bidentate cyclo­hexane-1,2-diamine ligand. In the crystal, pairs of mol­ecules form inversion dimers *via* N—H⋯Cl hydrogen bonds. In addition, intra­molecular N—H⋯Cl and weak C—H⋯Cl, C—H⋯N, N—H⋯π and C—H⋯π hydrogen bonds are observed. One of the Cl atoms of the solvent mol­ecule is disordered over two sites with refined occupancies of 0.62 (1) and 0.38 (1).

## Related literature

For the coordination chemistry of ruthenium complexes and their applications, see: Lindner, Mayer *et al.* (2003 ▶); Noyori (1994 ▶, 2003 ▶); Ohkuma *et al.* (2002 ▶); Lindner *et al.* (2005 ▶); Noyori & Ohkuma (2001 ▶); Lindner, Warad *et al.* (2003 ▶). For evidence of intra- and inter­molecular inter­actions in similar complexes, see: Warad (2007 ▶, 2010 ▶). 
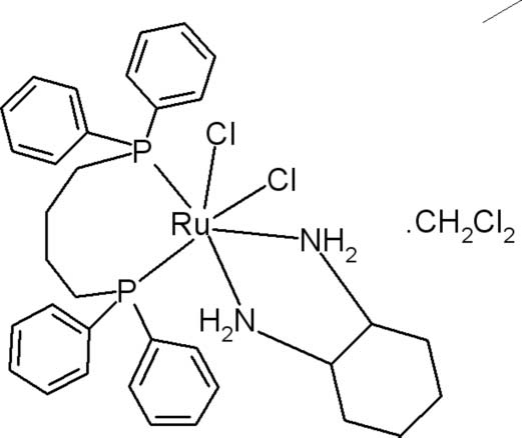


## Experimental

### 

#### Crystal data

[RuCl_2_(C_6_H_14_N_2_)(C_28_H_28_P_2_)]·CH_2_Cl_2_
*M* *_r_* = 797.53
Monoclinic, 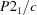

*a* = 12.419 (7) Å
*b* = 19.722 (10) Å
*c* = 17.588 (7) Å
β = 123.25 (3)°
*V* = 3603 (3) Å^3^
*Z* = 4
Mo *K*α radiation
μ = 0.85 mm^−1^
*T* = 296 K
0.28 × 0.17 × 0.09 mm

#### Data collection

Enraf–Nonius CAD-4 diffractometer
9791 measured reflections
8141 independent reflections
6777 reflections with *I* > 2σ(*I*)
*R*_int_ = 0.028
2 standard reflections every 120 min intensity decay: none

#### Refinement

*R*[*F* ^2^ > 2σ(*F* ^2^)] = 0.050
*wR*(*F* ^2^) = 0.135
*S* = 1.03
8141 reflections
413 parameters
5 restraints
H atoms treated by a mixture of independent and constrained refinement
Δρ_max_ = 0.95 e Å^−3^
Δρ_min_ = −0.97 e Å^−3^

### 

Data collection: *CAD-4 EXPRESS* (Enraf–Nonius, 1994 ▶); cell refinement: *CAD-4 EXPRESS*; data reduction: *XCAD4* (Harms & Wocadlo, 1995 ▶); program(s) used to solve structure: *SHELXS97* (Sheldrick, 2008 ▶); program(s) used to refine structure: *SHELXL97* (Sheldrick, 2008 ▶); molecular graphics: *ORTEP-3 for Windows* (Farrugia, 1997 ▶) and *DIAMOND* (Brandenburg & Putz, 2005 ▶); software used to prepare material for publication: *WinGX* (Farrugia, 1999 ▶).

## Supplementary Material

Crystal structure: contains datablock(s) I, global. DOI: 10.1107/S1600536812014080/lh5442sup1.cif

Structure factors: contains datablock(s) I. DOI: 10.1107/S1600536812014080/lh5442Isup2.hkl

Additional supplementary materials:  crystallographic information; 3D view; checkCIF report

## Figures and Tables

**Table 1:** Hydrogen-bond geometry (Å, °) *Cg*1 and *Cg*2 are the centroids of the C1–C6 and C22–C27 rings, respectively.

*D*—H⋯*A*	*D*—H	H⋯*A*	*D*⋯*A*	*D*—H⋯*A*
N1—H1*A*⋯Cl2	0.85 (1)	2.40 (3)	2.983 (4)	125 (3)
N2—H2*B*⋯Cl1^i^	0.90	2.62	3.390 (3)	143
C3—H3*A*⋯Cl2^i^	0.93	2.80	3.601 (5)	144
C9—H9*A*⋯Cl4*A*^ii^	0.93	2.83	3.492 (9)	130
C12—H12*A*⋯N1	0.93	2.51	3.305 (5)	144
C13—H13*A*⋯Cl1	0.97	2.76	3.383 (5)	123
C18—H18*A*⋯Cl1	0.93	2.76	3.499 (5)	137
C18—H18*A*⋯Cl2	0.93	2.79	3.361 (5)	121
C35*A*—H35*A*⋯Cl2^i^	0.97	2.57	3.520 (8)	167
N2—H2*A*⋯*Cg*1	0.90	2.74	3.612	164
C26—H26*A*⋯*Cg*2^iii^	0.93	2.78	3.577	142

Symmetry codes: (i) 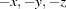; (ii) 
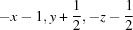
; (iii) 
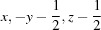
.

**Fig. 1.**
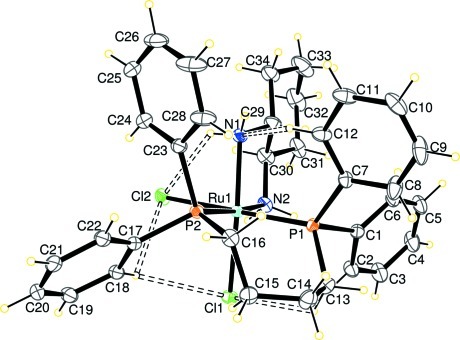
An ORTEP (Farrugia, 1997) view of the title complex. Displacement ellipsoids are drawn at the 30% probability level. H atoms are represented as small spheres of arbitrary radii. Hydrogen bonds are shown as dashed lines.

**Fig. 2.**
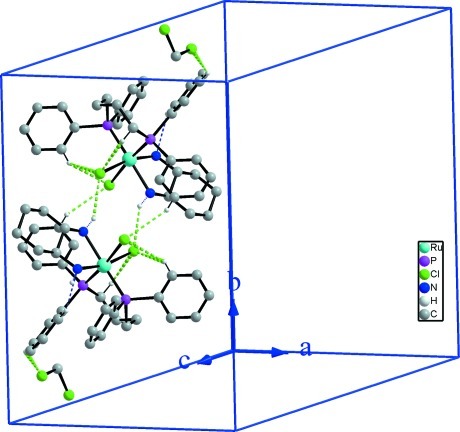
A view of the crystal packing of (I). The H-atoms not involved in H-bonding have been omitted. Hydrogen bonds are shown as dashed lines.

## supplementary crystallographic information

### Comment

Diphosphine compounds as chelate ligands have played a very important role in the design and development of metal complex-mediated catalysis (Lindner, Mayer *et al.*, 2003; Noyori, 1994, 2003). The chelating effects of diphosphine ligands decreases the number of isomers in complexes which decomplicates their structures (Ohkuma *et al.*, 2002; Lindner *et al.*, 2005). Many mixed diamine/diphosphine/ruthenium(II) complexes have been synthesized and characterized for their applications in the field of asymmetrical catalytic hydrogenation, photolysis and bioinorganic chemistry (Noyori & Ohkuma, 2001; Lindner, Warad *et al.*, 2003).

In this work, we report the synthesis and crystal structure of the title complex. The complex *cis*-[RuCl_2_(chd)(dppb)] is in full *cis* form (Fig. 1) with a solvent molecule of dichloromethane. The Ru^II^ ion is in a slightly distorted octahedral environment with a five-membered (chd) ring coordinating *via* N1 and N2, a seven-membered (dppb) ring coordinating *via* P1 and P2 as well as two Cl atoms. In the seven-membered ring of dppb the P—Ru—P angle is larger than the ideal value for perfectly octahedral. The 1,2-cyclohexanediamine ring also enforces distortion of the N—Ru—N angle [79.89 (13)°] while the Cl–Ru–Cl angle is closer to ideal [90.73 (4)°]. One Cl atom of the CH_2_Cl_2_ solvent is disordered over two positions with a site-occupancy ratio of 0.62 (1):0.38 (1). The molecular conformation and the crystal packing show various intra and intermolecular contacts of the types N–H···Cl, C–H···Cl and C–H···N (Table 1 and Fig. 2). The molecule and crystal structure are further stabilized by intramolecular N–H···π and intermolecular C–H···π interactions (Table 1). The values of these interactions are similar to those observed in other ruthenium complexes of the same type (Warad, 2007, 2010).

### Experimental

1,2-cyclohexanediamine (0.21 mmol) was dissolved in 10 ml of dry dichloromethane and the resultant solution was added drop-wise to a stirred solution of (RuCl_2_(dppb)PPh_3_) complex (0.20 mmol) dissolved in 10 ml of dry dichloromethane. The reaction mixture was stirred for 5 min at room temperature under inert atmosphere resulting in a change in color from green to light yellow. The resulting yellow solution was concentrated by vacuum to 1 ml followed by addition of 30 ml of diethyl ether to cause desired complex formation as precipitation. The resulting precipitate was collected and recrystallized from dichloromethane/diethyl ether and obtained in analytically pure form.

### Refinement

All H atoms attached to C and N atoms except those attached to N1 atom, were fixed geometrically and treated as riding, with C—H = 0.93-0.98 Å and N—H = 0.90 Å and with *U*_iso_(H) = 1.2U_eq_(C or N). The H atoms bonded to N1 were refined independently with U_iso_= 0.05Å^2^.

### Crystal data

**Table:**

[RuCl_2_(C_6_H_14_N_2_)(C_28_H_28_P_2_)]·CH_2_Cl_2_	*F*(000) = 1640
*M**_r_* = 797.53	*D*_x_ = 1.470 Mg m^−^^3^
Monoclinic, *P*2_1_/*c*	Mo *K*α radiation, λ = 0.71073 Å
Hall symbol: -P 2ybc	Cell parameters from 25 reflections
*a* = 12.419 (7) Å	θ = 9–11°
*b* = 19.722 (10) Å	µ = 0.85 mm^−^^1^
*c* = 17.588 (7) Å	*T* = 296 K
β = 123.25 (3)°	Prism, colorless
*V* = 3603 (3) Å^3^	0.28 × 0.17 × 0.09 mm
*Z* = 4	

### Data collection

**Table:**

Enraf–Nonius CAD-4 diffractometer	*R*_int_ = 0.028
Radiation source: fine-focus sealed tube	θ_max_ = 27.6°, θ_min_ = 2.2°
Graphite monochromator	*h* = −15→1
non–profiled ω scans	*k* = −25→1
9791 measured reflections	*l* = −19→22
8141 independent reflections	2 standard reflections every 120 min
6777 reflections with *I* > 2σ(*I*)	intensity decay: none

### Refinement

**Table:**

Refinement on *F*^2^	Primary atom site location: structure-invariant direct methods
Least-squares matrix: full	Secondary atom site location: difference Fourier map
*R*[*F*^2^ > 2σ(*F*^2^)] = 0.050	Hydrogen site location: inferred from neighbouring sites
*wR*(*F*^2^) = 0.135	H atoms treated by a mixture of independent and constrained refinement
*S* = 1.03	*w* = 1/[σ^2^(*F*_o_^2^) + (0.0616*P*)^2^ + 6.8898*P*] where *P* = (*F*_o_^2^ + 2*F*_c_^2^)/3
8141 reflections	(Δ/σ)_max_ < 0.001
413 parameters	Δρ_max_ = 0.95 e Å^−^^3^
5 restraints	Δρ_min_ = −0.97 e Å^−^^3^

### Special details

**Table:**

Geometry. All e.s.d.'s (except the e.s.d. in the dihedral angle between two l.s. planes) are estimated using the full covariance matrix. The cell e.s.d.'s are taken into account individually in the estimation of e.s.d.'s in distances, angles and torsion angles; correlations between e.s.d.'s in cell parameters are only used when they are defined by crystal symmetry. An approximate (isotropic) treatment of cell e.s.d.'s is used for estimating e.s.d.'s involving l.s. planes.
Refinement. Refinement of *F*^2^ against ALL reflections. The weighted *R*-factor *wR* and goodness of fit *S* are based on *F*^2^, conventional *R*-factors *R* are based on *F*, with *F* set to zero for negative *F*^2^. The threshold expression of *F*^2^ > σ(*F*^2^) is used only for calculating *R*-factors(gt) *etc*. and is not relevant to the choice of reflections for refinement. *R*-factors based on *F*^2^ are statistically about twice as large as those based on *F*, and *R*- factors based on ALL data will be even larger.

### Fractional atomic coordinates and isotropic or equivalent isotropic displacement parameters (Å^2^)

**Table:**

	*x*	*y*	*z*	*U*_iso_*/*U*_eq_	Occ. (<1)
Ru1	0.09441 (3)	0.117180 (13)	0.157004 (17)	0.02961 (9)	
Cl1	0.08731 (10)	0.10589 (5)	0.01660 (6)	0.0414 (2)	
Cl2	0.25788 (10)	0.02474 (5)	0.22871 (7)	0.0479 (2)	
P1	−0.07122 (9)	0.19245 (4)	0.08538 (6)	0.03276 (19)	
P2	0.24807 (9)	0.19891 (5)	0.21823 (6)	0.03282 (19)	
N1	0.0960 (3)	0.10147 (17)	0.2766 (2)	0.0420 (7)	
H1A	0.166 (2)	0.0786 (17)	0.303 (2)	0.050*	
H1B	0.106 (3)	0.1333 (10)	0.3119 (14)	0.050*	
N2	−0.0355 (3)	0.03164 (15)	0.1149 (2)	0.0402 (7)	
H2A	−0.1149	0.0443	0.0693	0.048*	
H2B	−0.0086	−0.0017	0.0942	0.048*	
C1	−0.2286 (4)	0.14977 (19)	0.0157 (2)	0.0386 (8)	
C2	−0.2516 (4)	0.1105 (2)	−0.0582 (3)	0.0510 (10)	
H2C	−0.1902	0.1089	−0.0729	0.061*	
C3	−0.3651 (4)	0.0737 (3)	−0.1098 (3)	0.0662 (14)	
H3A	−0.3791	0.0479	−0.1587	0.079*	
C4	−0.4553 (4)	0.0753 (3)	−0.0893 (4)	0.0680 (14)	
H4A	−0.5311	0.0508	−0.1244	0.082*	
C5	−0.4353 (4)	0.1130 (3)	−0.0169 (4)	0.0600 (12)	
H5A	−0.4975	0.1137	−0.0029	0.072*	
C6	−0.3225 (4)	0.1500 (2)	0.0354 (3)	0.0474 (9)	
H6A	−0.3098	0.1753	0.0844	0.057*	
C7	−0.1044 (4)	0.25398 (18)	0.1491 (3)	0.0393 (8)	
C8	−0.2028 (5)	0.3004 (3)	0.1063 (4)	0.0697 (15)	
H8A	−0.2564	0.3008	0.0432	0.084*	
C9	−0.2226 (5)	0.3467 (3)	0.1572 (5)	0.0783 (18)	
H9A	−0.2889	0.3782	0.1274	0.094*	
C10	−0.1476 (5)	0.3469 (2)	0.2489 (4)	0.0619 (14)	
H10A	−0.1637	0.3776	0.2818	0.074*	
C11	−0.0477 (6)	0.3017 (2)	0.2936 (3)	0.0613 (13)	
H11A	0.0062	0.3022	0.3567	0.074*	
C12	−0.0286 (5)	0.2551 (2)	0.2427 (3)	0.0477 (10)	
H12A	0.0376	0.2237	0.2728	0.057*	
C13	−0.0866 (4)	0.2454 (2)	−0.0064 (3)	0.0461 (9)	
H13A	−0.0779	0.2156	−0.0466	0.055*	
H13B	−0.1739	0.2629	−0.0411	0.055*	
C14	0.0028 (5)	0.3051 (2)	0.0151 (3)	0.0568 (11)	
H14A	−0.0133	0.3385	0.0482	0.068*	
H14B	−0.0193	0.3258	−0.0418	0.068*	
C15	0.1463 (4)	0.2893 (2)	0.0701 (3)	0.0483 (9)	
H15A	0.1911	0.3243	0.0592	0.058*	
H15B	0.1602	0.2465	0.0495	0.058*	
C16	0.2036 (4)	0.28476 (19)	0.1728 (3)	0.0419 (8)	
H16A	0.1412	0.3023	0.1847	0.050*	
H16B	0.2794	0.3134	0.2047	0.050*	
C17	0.3984 (4)	0.1885 (2)	0.2220 (2)	0.0413 (8)	
C18	0.4145 (4)	0.1354 (2)	0.1772 (3)	0.0475 (9)	
H18A	0.3490	0.1040	0.1442	0.057*	
C19	0.5304 (5)	0.1299 (3)	0.1827 (3)	0.0641 (14)	
H19A	0.5423	0.0938	0.1539	0.077*	
C20	0.6273 (5)	0.1765 (4)	0.2294 (3)	0.0729 (17)	
H20A	0.7038	0.1721	0.2319	0.087*	
C21	0.6104 (4)	0.2297 (4)	0.2724 (3)	0.0707 (16)	
H21A	0.6750	0.2619	0.3032	0.085*	
C22	0.4981 (4)	0.2353 (3)	0.2698 (3)	0.0551 (11)	
H22A	0.4884	0.2708	0.3004	0.066*	
C23	0.3157 (4)	0.2116 (2)	0.3403 (3)	0.0450 (9)	
C24	0.3864 (5)	0.1601 (4)	0.4000 (3)	0.0715 (16)	
H24A	0.4048	0.1214	0.3789	0.086*	
C25	0.4309 (5)	0.1654 (4)	0.4920 (3)	0.085 (2)	
H25A	0.4811	0.1310	0.5321	0.102*	
C26	0.4006 (6)	0.2211 (4)	0.5231 (3)	0.081 (2)	
H26A	0.4277	0.2240	0.5839	0.097*	
C27	0.3314 (7)	0.2712 (3)	0.4653 (4)	0.083 (2)	
H27A	0.3115	0.3091	0.4867	0.100*	
C28	0.2889 (5)	0.2678 (2)	0.3741 (3)	0.0611 (13)	
H28A	0.2419	0.3036	0.3355	0.073*	
C29	−0.0180 (5)	0.0627 (2)	0.2553 (3)	0.0558 (11)	
H29A	−0.0920	0.0934	0.2229	0.067*	
C30	−0.0406 (5)	0.0062 (2)	0.1918 (4)	0.0569 (11)	
H30A	0.0323	−0.0249	0.2256	0.068*	
C31	−0.1612 (5)	−0.0348 (2)	0.1633 (4)	0.0633 (13)	
H31A	−0.2371	−0.0071	0.1255	0.076*	
H31B	−0.1661	−0.0738	0.1280	0.076*	
C32	−0.1579 (7)	−0.0581 (3)	0.2464 (5)	0.091 (2)	
H32A	−0.2399	−0.0790	0.2269	0.109*	
H32B	−0.0918	−0.0926	0.2773	0.109*	
C33	−0.1323 (8)	−0.0032 (4)	0.3119 (6)	0.100 (2)	
H33A	−0.1221	−0.0229	0.3660	0.120*	
H33B	−0.2059	0.0270	0.2850	0.120*	
C34	−0.0132 (5)	0.0374 (3)	0.3388 (4)	0.0661 (14)	
H34A	0.0626	0.0093	0.3751	0.079*	
H34B	−0.0065	0.0758	0.3757	0.079*	
Cl3	−0.6261 (2)	0.06177 (11)	−0.35466 (17)	0.1163 (7)	
Cl4A	−0.6581 (11)	−0.0287 (6)	−0.4888 (4)	0.362 (13)	0.620 (10)
Cl4B	−0.7233 (6)	−0.0653 (3)	−0.4351 (5)	0.122 (3)	0.380 (10)
C35A	−0.5949 (7)	−0.0161 (4)	−0.3781 (6)	0.122 (3)	
H35A	−0.5024	−0.0224	−0.3450	0.146*	0.620 (10)
H35B	−0.6289	−0.0500	−0.3566	0.146*	0.620 (10)
H35C	−0.5564	−0.0111	−0.4131	0.146*	0.380 (10)
H35D	−0.5323	−0.0383	−0.3213	0.146*	0.380 (10)

### Atomic displacement parameters (Å^2^)

**Table:**

	*U*^11^	*U*^22^	*U*^33^	*U*^12^	*U*^13^	*U*^23^
Ru1	0.03435 (15)	0.02501 (14)	0.03072 (14)	0.00161 (10)	0.01864 (12)	−0.00178 (10)
Cl1	0.0523 (5)	0.0394 (4)	0.0376 (4)	−0.0056 (4)	0.0280 (4)	−0.0104 (3)
Cl2	0.0515 (6)	0.0389 (5)	0.0629 (6)	0.0154 (4)	0.0376 (5)	0.0105 (4)
P1	0.0346 (4)	0.0312 (4)	0.0287 (4)	0.0030 (3)	0.0149 (4)	−0.0027 (3)
P2	0.0328 (4)	0.0365 (4)	0.0302 (4)	−0.0034 (3)	0.0179 (4)	−0.0055 (3)
N1	0.0481 (19)	0.0428 (17)	0.0407 (17)	0.0090 (14)	0.0279 (15)	0.0071 (13)
N2	0.0461 (18)	0.0267 (14)	0.0563 (19)	−0.0031 (12)	0.0334 (16)	−0.0053 (13)
C1	0.0381 (19)	0.0397 (19)	0.0343 (17)	0.0006 (15)	0.0175 (15)	−0.0078 (14)
C2	0.041 (2)	0.064 (3)	0.047 (2)	−0.0062 (19)	0.0234 (18)	−0.0206 (19)
C3	0.047 (2)	0.081 (3)	0.062 (3)	−0.009 (2)	0.024 (2)	−0.038 (3)
C4	0.036 (2)	0.080 (4)	0.074 (3)	−0.012 (2)	0.022 (2)	−0.031 (3)
C5	0.037 (2)	0.075 (3)	0.069 (3)	−0.004 (2)	0.029 (2)	−0.018 (2)
C6	0.039 (2)	0.054 (2)	0.044 (2)	0.0042 (17)	0.0196 (17)	−0.0126 (18)
C7	0.0379 (19)	0.0327 (17)	0.0446 (19)	0.0001 (14)	0.0209 (16)	−0.0083 (15)
C8	0.053 (3)	0.061 (3)	0.064 (3)	0.020 (2)	0.012 (2)	−0.021 (2)
C9	0.051 (3)	0.053 (3)	0.111 (5)	0.014 (2)	0.032 (3)	−0.025 (3)
C10	0.074 (3)	0.039 (2)	0.103 (4)	−0.019 (2)	0.068 (3)	−0.032 (2)
C11	0.107 (4)	0.038 (2)	0.063 (3)	−0.006 (2)	0.062 (3)	−0.011 (2)
C12	0.073 (3)	0.0336 (18)	0.048 (2)	0.0026 (18)	0.041 (2)	−0.0034 (16)
C13	0.046 (2)	0.046 (2)	0.0369 (19)	0.0057 (17)	0.0170 (17)	0.0095 (16)
C14	0.059 (3)	0.045 (2)	0.059 (3)	0.004 (2)	0.027 (2)	0.017 (2)
C15	0.053 (2)	0.040 (2)	0.054 (2)	−0.0015 (18)	0.031 (2)	0.0070 (17)
C16	0.044 (2)	0.0339 (18)	0.049 (2)	−0.0071 (15)	0.0264 (18)	−0.0071 (15)
C17	0.0355 (19)	0.058 (2)	0.0319 (17)	0.0004 (16)	0.0193 (15)	0.0049 (16)
C18	0.048 (2)	0.059 (2)	0.043 (2)	0.0144 (19)	0.0295 (19)	0.0114 (18)
C19	0.063 (3)	0.087 (4)	0.059 (3)	0.027 (3)	0.044 (3)	0.025 (3)
C20	0.038 (2)	0.135 (5)	0.051 (3)	0.017 (3)	0.027 (2)	0.027 (3)
C21	0.034 (2)	0.135 (5)	0.035 (2)	−0.014 (3)	0.0141 (18)	0.002 (3)
C22	0.038 (2)	0.089 (3)	0.037 (2)	−0.013 (2)	0.0195 (17)	−0.007 (2)
C23	0.0346 (19)	0.066 (3)	0.0338 (18)	−0.0139 (18)	0.0185 (16)	−0.0112 (17)
C24	0.051 (3)	0.122 (5)	0.039 (2)	0.025 (3)	0.023 (2)	0.007 (3)
C25	0.048 (3)	0.163 (7)	0.037 (2)	0.013 (3)	0.019 (2)	0.014 (3)
C26	0.072 (3)	0.138 (6)	0.040 (2)	−0.046 (4)	0.036 (3)	−0.030 (3)
C27	0.133 (6)	0.078 (4)	0.064 (3)	−0.045 (4)	0.071 (4)	−0.033 (3)
C28	0.095 (4)	0.051 (2)	0.051 (2)	−0.028 (2)	0.049 (3)	−0.021 (2)
C29	0.072 (3)	0.046 (2)	0.070 (3)	0.006 (2)	0.052 (3)	0.008 (2)
C30	0.072 (3)	0.037 (2)	0.079 (3)	−0.001 (2)	0.052 (3)	0.007 (2)
C31	0.065 (3)	0.038 (2)	0.108 (4)	0.001 (2)	0.061 (3)	0.008 (2)
C32	0.103 (5)	0.058 (3)	0.160 (7)	0.009 (3)	0.102 (5)	0.035 (4)
C33	0.137 (6)	0.082 (4)	0.151 (6)	0.024 (4)	0.123 (6)	0.040 (4)
C34	0.086 (4)	0.060 (3)	0.079 (3)	0.021 (3)	0.062 (3)	0.027 (3)
Cl3	0.1221 (16)	0.1016 (14)	0.1364 (17)	0.0333 (12)	0.0780 (14)	0.0096 (12)
Cl4A	0.335 (12)	0.317 (12)	0.119 (5)	0.249 (11)	−0.076 (7)	−0.104 (7)
Cl4B	0.128 (5)	0.099 (4)	0.078 (4)	−0.016 (3)	0.018 (3)	−0.020 (3)
C35A	0.079 (5)	0.085 (5)	0.150 (7)	0.025 (4)	0.030 (5)	0.001 (5)
C35B	0.079 (5)	0.085 (5)	0.150 (7)	0.025 (4)	0.030 (5)	0.001 (5)

### Geometric parameters (Å, º)

**Table:**

Ru1—N1	2.115 (3)	C15—H15A	0.9700
Ru1—N2	2.165 (3)	C15—H15B	0.9700
Ru1—P2	2.2684 (13)	C16—H16A	0.9700
Ru1—P1	2.2761 (13)	C16—H16B	0.9700
Ru1—Cl1	2.4325 (13)	C17—C18	1.389 (6)
Ru1—Cl2	2.4956 (14)	C17—C22	1.397 (6)
P1—C13	1.843 (4)	C18—C19	1.393 (6)
P1—C1	1.845 (4)	C18—H18A	0.9300
P1—C7	1.845 (4)	C19—C20	1.372 (8)
P2—C16	1.824 (4)	C19—H19A	0.9300
P2—C17	1.843 (4)	C20—C21	1.376 (9)
P2—C23	1.844 (4)	C20—H20A	0.9300
N1—C29	1.466 (6)	C21—C22	1.375 (6)
N1—H1A	0.854 (10)	C21—H21A	0.9300
N1—H1B	0.843 (10)	C22—H22A	0.9300
N2—C30	1.475 (5)	C23—C24	1.376 (7)
N2—H2A	0.9000	C23—C28	1.382 (6)
N2—H2B	0.9000	C24—C25	1.398 (6)
C1—C6	1.387 (6)	C24—H24A	0.9300
C1—C2	1.402 (5)	C25—C26	1.369 (9)
C2—C3	1.390 (6)	C25—H25A	0.9300
C2—H2C	0.9300	C26—C27	1.338 (9)
C3—C4	1.352 (7)	C26—H26A	0.9300
C3—H3A	0.9300	C27—C28	1.387 (7)
C4—C5	1.375 (7)	C27—H27A	0.9300
C4—H4A	0.9300	C28—H28A	0.9300
C5—C6	1.388 (6)	C29—C30	1.492 (7)
C5—H5A	0.9300	C29—C34	1.520 (6)
C6—H6A	0.9300	C29—H29A	0.9800
C7—C8	1.374 (6)	C30—C31	1.525 (6)
C7—C12	1.377 (6)	C30—H30A	0.9800
C8—C9	1.392 (7)	C31—C32	1.510 (8)
C8—H8A	0.9300	C31—H31A	0.9700
C9—C10	1.350 (8)	C31—H31B	0.9700
C9—H9A	0.9300	C32—C33	1.484 (10)
C10—C11	1.372 (7)	C32—H32A	0.9700
C10—H10A	0.9300	C32—H32B	0.9700
C11—C12	1.391 (5)	C33—C34	1.512 (9)
C11—H11A	0.9300	C33—H33A	0.9700
C12—H12A	0.9300	C33—H33B	0.9700
C13—C14	1.517 (6)	C34—H34A	0.9700
C13—H13A	0.9700	C34—H34B	0.9700
C13—H13B	0.9700	Cl3—C35A	1.689 (8)
C14—C15	1.522 (6)	Cl4A—C35A	1.672 (9)
C14—H14A	0.9700	C35A—H35A	0.9700
C14—H14B	0.9700	C35A—H35B	0.9700
C15—C16	1.539 (6)		
			
N1—Ru1—N2	79.90 (13)	C16—C15—H15A	109.1
N1—Ru1—P2	94.89 (10)	C14—C15—H15B	109.1
N2—Ru1—P2	172.40 (9)	C16—C15—H15B	109.1
N1—Ru1—P1	99.14 (10)	H15A—C15—H15B	107.8
N2—Ru1—P1	92.35 (10)	C15—C16—P2	113.7 (3)
P2—Ru1—P1	93.96 (6)	C15—C16—H16A	108.8
N1—Ru1—Cl1	166.27 (10)	P2—C16—H16A	108.8
N2—Ru1—Cl1	88.65 (9)	C15—C16—H16B	108.8
P2—Ru1—Cl1	95.72 (4)	P2—C16—H16B	108.8
P1—Ru1—Cl1	88.80 (5)	H16A—C16—H16B	107.7
N1—Ru1—Cl2	80.15 (10)	C18—C17—C22	118.9 (4)
N2—Ru1—Cl2	81.42 (10)	C18—C17—P2	121.5 (3)
P2—Ru1—Cl2	92.28 (6)	C22—C17—P2	119.6 (3)
P1—Ru1—Cl2	173.76 (4)	C17—C18—C19	119.0 (5)
Cl1—Ru1—Cl2	90.73 (4)	C17—C18—H18A	120.5
C13—P1—C1	96.53 (18)	C19—C18—H18A	120.5
C13—P1—C7	102.05 (19)	C20—C19—C18	121.6 (5)
C1—P1—C7	101.27 (17)	C20—C19—H19A	119.2
C13—P1—Ru1	118.85 (15)	C18—C19—H19A	119.2
C1—P1—Ru1	112.13 (13)	C19—C20—C21	119.4 (4)
C7—P1—Ru1	121.88 (13)	C19—C20—H20A	120.3
C16—P2—C17	100.21 (19)	C21—C20—H20A	120.3
C16—P2—C23	102.6 (2)	C22—C21—C20	120.1 (5)
C17—P2—C23	99.64 (17)	C22—C21—H21A	120.0
C16—P2—Ru1	118.54 (13)	C20—C21—H21A	120.0
C17—P2—Ru1	120.63 (14)	C21—C22—C17	121.0 (5)
C23—P2—Ru1	112.16 (13)	C21—C22—H22A	119.5
C29—N1—Ru1	110.0 (3)	C17—C22—H22A	119.5
C29—N1—H1A	114 (3)	C24—C23—C28	117.9 (4)
Ru1—N1—H1A	94 (3)	C24—C23—P2	118.7 (4)
C29—N1—H1B	108 (3)	C28—C23—P2	123.1 (4)
Ru1—N1—H1B	123 (2)	C23—C24—C25	120.7 (6)
H1A—N1—H1B	107.4 (16)	C23—C24—H24A	119.7
C30—N2—Ru1	110.5 (3)	C25—C24—H24A	119.7
C30—N2—H2A	109.6	C26—C25—C24	120.1 (6)
Ru1—N2—H2A	109.6	C26—C25—H25A	120.0
C30—N2—H2B	109.6	C24—C25—H25A	120.0
Ru1—N2—H2B	109.6	C27—C26—C25	119.4 (5)
H2A—N2—H2B	108.1	C27—C26—H26A	120.3
C6—C1—C2	117.5 (4)	C25—C26—H26A	120.3
C6—C1—P1	124.2 (3)	C26—C27—C28	121.5 (6)
C2—C1—P1	118.1 (3)	C26—C27—H27A	119.2
C3—C2—C1	120.8 (4)	C28—C27—H27A	119.2
C3—C2—H2C	119.6	C23—C28—C27	120.4 (5)
C1—C2—H2C	119.6	C23—C28—H28A	119.8
C4—C3—C2	120.4 (4)	C27—C28—H28A	119.8
C4—C3—H3A	119.8	N1—C29—C30	109.8 (4)
C2—C3—H3A	119.8	N1—C29—C34	113.8 (4)
C3—C4—C5	120.3 (4)	C30—C29—C34	111.8 (4)
C3—C4—H4A	119.9	N1—C29—H29A	107.0
C5—C4—H4A	119.9	C30—C29—H29A	107.0
C4—C5—C6	120.2 (4)	C34—C29—H29A	107.0
C4—C5—H5A	119.9	N2—C30—C29	110.4 (3)
C6—C5—H5A	119.9	N2—C30—C31	114.1 (4)
C1—C6—C5	120.9 (4)	C29—C30—C31	112.9 (4)
C1—C6—H6A	119.6	N2—C30—H30A	106.3
C5—C6—H6A	119.6	C29—C30—H30A	106.3
C8—C7—C12	117.6 (4)	C31—C30—H30A	106.3
C8—C7—P1	122.3 (3)	C32—C31—C30	110.1 (5)
C12—C7—P1	120.2 (3)	C32—C31—H31A	109.6
C7—C8—C9	120.2 (5)	C30—C31—H31A	109.6
C7—C8—H8A	119.9	C32—C31—H31B	109.6
C9—C8—H8A	119.9	C30—C31—H31B	109.6
C10—C9—C8	121.4 (5)	H31A—C31—H31B	108.2
C10—C9—H9A	119.3	C33—C32—C31	114.1 (5)
C8—C9—H9A	119.3	C33—C32—H32A	108.7
C9—C10—C11	119.8 (4)	C31—C32—H32A	108.7
C9—C10—H10A	120.1	C33—C32—H32B	108.7
C11—C10—H10A	120.1	C31—C32—H32B	108.7
C10—C11—C12	118.7 (5)	H32A—C32—H32B	107.6
C10—C11—H11A	120.7	C32—C33—C34	112.7 (5)
C12—C11—H11A	120.7	C32—C33—H33A	109.0
C7—C12—C11	122.3 (4)	C34—C33—H33A	109.0
C7—C12—H12A	118.8	C32—C33—H33B	109.0
C11—C12—H12A	118.8	C34—C33—H33B	109.0
C14—C13—P1	120.7 (3)	H33A—C33—H33B	107.8
C14—C13—H13A	107.1	C33—C34—C29	110.9 (5)
P1—C13—H13A	107.1	C33—C34—H34A	109.5
C14—C13—H13B	107.1	C29—C34—H34A	109.5
P1—C13—H13B	107.1	C33—C34—H34B	109.5
H13A—C13—H13B	106.8	C29—C34—H34B	109.5
C13—C14—C15	116.1 (4)	H34A—C34—H34B	108.0
C13—C14—H14A	108.2	Cl4A—C35A—Cl3	113.2 (5)
C15—C14—H14A	108.2	Cl4A—C35A—H35A	108.9
C13—C14—H14B	108.2	Cl3—C35A—H35A	108.9
C15—C14—H14B	108.2	Cl4A—C35A—H35B	108.9
H14A—C14—H14B	107.4	Cl3—C35A—H35B	108.9
C14—C15—C16	112.6 (4)	H35A—C35A—H35B	107.7
C14—C15—H15A	109.1		
			
N1—Ru1—P1—C13	160.98 (18)	C9—C10—C11—C12	−1.9 (7)
N2—Ru1—P1—C13	−118.88 (18)	C8—C7—C12—C11	−1.0 (7)
P2—Ru1—P1—C13	65.37 (16)	P1—C7—C12—C11	178.3 (4)
Cl1—Ru1—P1—C13	−30.29 (16)	C10—C11—C12—C7	1.7 (7)
N1—Ru1—P1—C1	−87.66 (16)	C1—P1—C13—C14	165.2 (4)
N2—Ru1—P1—C1	−7.52 (15)	C7—P1—C13—C14	62.2 (4)
P2—Ru1—P1—C1	176.73 (13)	Ru1—P1—C13—C14	−75.0 (4)
Cl1—Ru1—P1—C1	81.08 (13)	P1—C13—C14—C15	56.9 (5)
N1—Ru1—P1—C7	32.42 (18)	C13—C14—C15—C16	−81.9 (5)
N2—Ru1—P1—C7	112.57 (17)	C14—C15—C16—P2	109.5 (4)
P2—Ru1—P1—C7	−63.18 (15)	C17—P2—C16—C15	76.2 (3)
Cl1—Ru1—P1—C7	−158.84 (15)	C23—P2—C16—C15	178.6 (3)
N1—Ru1—P2—C16	−114.72 (17)	Ru1—P2—C16—C15	−57.3 (3)
P1—Ru1—P2—C16	−15.18 (15)	C16—P2—C17—C18	−123.4 (3)
Cl1—Ru1—P2—C16	74.01 (15)	C23—P2—C17—C18	131.7 (3)
Cl2—Ru1—P2—C16	164.98 (15)	Ru1—P2—C17—C18	8.7 (4)
N1—Ru1—P2—C17	121.46 (17)	C16—P2—C17—C22	55.7 (4)
P1—Ru1—P2—C17	−139.01 (14)	C23—P2—C17—C22	−49.1 (4)
Cl1—Ru1—P2—C17	−49.81 (14)	Ru1—P2—C17—C22	−172.1 (3)
Cl2—Ru1—P2—C17	41.16 (14)	C22—C17—C18—C19	1.0 (6)
N1—Ru1—P2—C23	4.63 (18)	P2—C17—C18—C19	−179.8 (3)
P1—Ru1—P2—C23	104.16 (16)	C17—C18—C19—C20	−1.4 (7)
Cl1—Ru1—P2—C23	−166.64 (16)	C18—C19—C20—C21	0.3 (7)
Cl2—Ru1—P2—C23	−75.67 (16)	C19—C20—C21—C22	1.2 (8)
N2—Ru1—N1—C29	−20.2 (3)	C20—C21—C22—C17	−1.7 (7)
P2—Ru1—N1—C29	165.4 (3)	C18—C17—C22—C21	0.5 (7)
P1—Ru1—N1—C29	70.6 (3)	P2—C17—C22—C21	−178.7 (4)
Cl1—Ru1—N1—C29	−54.1 (5)	C16—P2—C23—C24	−165.3 (4)
Cl2—Ru1—N1—C29	−103.2 (3)	C17—P2—C23—C24	−62.4 (4)
N1—Ru1—N2—C30	−5.7 (3)	Ru1—P2—C23—C24	66.4 (4)
P1—Ru1—N2—C30	−104.5 (3)	C16—P2—C23—C28	21.1 (4)
Cl1—Ru1—N2—C30	166.7 (3)	C17—P2—C23—C28	124.0 (4)
Cl2—Ru1—N2—C30	75.8 (3)	Ru1—P2—C23—C28	−107.2 (4)
C13—P1—C1—C6	−123.7 (4)	C28—C23—C24—C25	−0.9 (8)
C7—P1—C1—C6	−20.0 (4)	P2—C23—C24—C25	−174.9 (4)
Ru1—P1—C1—C6	111.5 (3)	C23—C24—C25—C26	2.3 (9)
C13—P1—C1—C2	61.1 (4)	C24—C25—C26—C27	−2.0 (9)
C7—P1—C1—C2	164.8 (3)	C25—C26—C27—C28	0.5 (9)
Ru1—P1—C1—C2	−63.7 (4)	C24—C23—C28—C27	−0.6 (7)
C6—C1—C2—C3	0.5 (7)	P2—C23—C28—C27	173.1 (4)
P1—C1—C2—C3	176.0 (4)	C26—C27—C28—C23	0.8 (9)
C1—C2—C3—C4	−0.1 (8)	Ru1—N1—C29—C30	43.0 (4)
C2—C3—C4—C5	−0.3 (9)	Ru1—N1—C29—C34	169.1 (3)
C3—C4—C5—C6	0.3 (9)	Ru1—N2—C30—C29	30.3 (5)
C2—C1—C6—C5	−0.5 (7)	Ru1—N2—C30—C31	158.6 (3)
P1—C1—C6—C5	−175.7 (4)	N1—C29—C30—N2	−48.7 (5)
C4—C5—C6—C1	0.1 (8)	C34—C29—C30—N2	−176.0 (4)
C13—P1—C7—C8	43.3 (4)	N1—C29—C30—C31	−177.7 (4)
C1—P1—C7—C8	−56.0 (4)	C34—C29—C30—C31	55.1 (6)
Ru1—P1—C7—C8	178.9 (4)	N2—C30—C31—C32	−179.7 (4)
C13—P1—C7—C12	−135.9 (3)	C29—C30—C31—C32	−52.6 (6)
C1—P1—C7—C12	124.8 (3)	C30—C31—C32—C33	51.3 (7)
Ru1—P1—C7—C12	−0.4 (4)	C31—C32—C33—C34	−52.4 (8)
C12—C7—C8—C9	0.5 (8)	C32—C33—C34—C29	52.3 (7)
P1—C7—C8—C9	−178.7 (4)	N1—C29—C34—C33	−178.9 (4)
C7—C8—C9—C10	−0.8 (9)	C30—C29—C34—C33	−53.8 (6)
C8—C9—C10—C11	1.5 (9)		

### Hydrogen-bond geometry (Å, º)

Cg1 and Cg2 are the centroids of the C1–C6 and C22–C27 rings, respectively.

**Table:**

*D*—H···*A*	*D*—H	H···*A*	*D*···*A*	*D*—H···*A*
N1—H1*A*···Cl2	0.85 (1)	2.40 (3)	2.983 (4)	125 (3)
N2—H2*B*···Cl1^i^	0.90	2.62	3.390 (3)	143
C3—H3*A*···Cl2^i^	0.93	2.80	3.601 (5)	144
C9—H9*A*···Cl4*A*^ii^	0.93	2.83	3.492 (9)	130
C12—H12*A*···N1	0.93	2.51	3.305 (5)	144
C13—H13*A*···Cl1	0.97	2.76	3.383 (5)	123
C18—H18*A*···Cl1	0.93	2.76	3.499 (5)	137
C18—H18*A*···Cl2	0.93	2.79	3.361 (5)	121
C35*A*—H35*A*···Cl2^i^	0.97	2.57	3.520 (8)	167
N2—H2*A*···*Cg*1	0.90	2.74	3.612	164
C26—H26*A*···*Cg*2^iii^	0.93	2.78	3.577	142

Symmetry codes: (i) −*x*, −*y*, −*z*; (ii) −*x*−1, *y*+1/2, −*z*−1/2; (iii) *x*, −*y*−1/2, *z*−1/2.
